# The Value of PET/CT-Based Radiomics in Predicting Adrenal Metastases in Patients with Cancer

**DOI:** 10.3390/diagnostics15111356

**Published:** 2025-05-28

**Authors:** Qiujun He, Xiangxing Kong, Xiangxi Meng, Xiuling Shen, Nan Li

**Affiliations:** 1 Department of Nuclear Medicine, Beijing Chest Hospital, Capital Medical University, Beijing Tuberculosis and Thoracic Tumor Research Institute, Beijing 101149, China; heqiujun0042@163.com; 2 State Key Laboratory of Holistic Integrative Management of Gastrointestinal Cancers, Beijing Key Laboratory of Carcinogenesis and Translational Research, National Medical Products Administration (NMPA) Key Laboratory for Research and Evaluation of Radiopharmaceuticals (National Medical Products Administration), Department of Nuclear Medicine, Peking University Cancer Hospital & Institute, Beijing 100142, China; 13563970213@163.com (X.K.); mengxiangxi@pku.edu.cn (X.M.); shenxiuling2022@163.com (X.S.)

**Keywords:** adrenal gland, metastatic tumor, FDG-PET, radiomics

## Abstract

**Objectives:** Differentiation of adrenal incidentalomas (AIs) remains a challenge in the oncological setting. The aim of the study was to explore the diagnostic efficacy of [18F]Fluorodeoxyglucose (FDG) positron emission tomography combined with computed tomography (PET/CT)-based radiomics in identifying adrenal metastases and to compare it with that of conventional PET/CT parameters. **Materials:** Retrospective analysis was performed on 195 AIs for model construction, nomogram drawing, and internal validation. An additional 30 AIs were collected for external validation of the radiomics model and nomogram. Logistic regression analysis was employed to build models based on clinical and PET/CT routine parameters. The open-source software Python (version 3.7.11) was utilized to process the regions of interest (ROI) delineated by ITK-SNAP, extracting radiomic features. Least absolute shrinkage and selection operator (LASSO) regression analysis was applied for feature selection. Based on the selected features, the optimal model was chosen from ten machine learning algorithms, and the nomogram was constructed. **Results:** The area under the curve (AUC), sensitivity, specificity, and accuracy of conventional parameters of PET/CT were 0.919, 0.849, 0.892, and 0.844, respectively. XGBoost demonstrated superior diagnostic efficiency among the radiomics models, outperforming those constructed using independent predictors. The AUC, accuracy, sensitivity, specificity, positive predictive value (PPV), and negative predictive value (NPV) of XGBoost’s internal and external validation were 0.945, 0.932, 0.930, 0.960, 0.970, 0.890 and 0.910, 0.900, 0.860, 1, 1, 0.750. The accuracy, sensitivity, specificity, PPV, and NPV of the nomogram in external validation were 0.870, 0.952, 0.667, 0.870, and 0.857. **Conclusions:** The radiomics model and conventional PET/CT parameters both showed high diagnostic performance (AUC *p* > 0.05) in discriminating adrenal metastases from benign lesions, offering a practical, non-invasive approach for clinical assessment.

## 1. Introduction

An adrenal incidentalomas (AI) is an adrenal mass with a diameter of >1 cm found during imaging examination accidentally without significant clinical manifestations [1,2]. Among AIs, adrenal adenomas are the most common benign tumors, and about 30% of adrenal adenomas are lipid-poor (>10 Hounsfield units [HU] on pre-enhanced CT value [3]. Adrenal metastasis is the second most common tumor after adrenal adenoma and also the most common malignancy of the adrenal gland [4]. In the population with known malignant tumors, metastatic lesions constitute a significantly high proportion of adrenal incidentalomas, ranging from 50% to 70% [5,6]. Resection of metastatic lesions can potentially improve survival rates in cancer patients under specific conditions [7]. Thus, precise qualitative diagnosis of adrenal incidentalomas in cancer patients during staging or follow-up is crucial for guiding treatment and predicting prognosis.

Currently, CT, MRI, positron emission tomography combined with computed tomography (PET/CT), and histopathological examination are the primary methods for determining the nature of adrenal incidentalomas. Due to the intrinsic fat content, chemical-shift MRI and unenhanced CT can effectively differentiate between benign and primary malignant lesions [8,9,10]. However, in patients with a history of extrarenal malignancies, these techniques exhibit a false negative rate of approximately 7% [11]. On contrast-enhanced CT, rapid washout is characteristic of adrenal adenomas [12,13], but this feature may overlap with angiomatous metastases, leading to potential diagnostic challenges [14,15]. PET/CT is increasingly recognized as a valuable tool for differentiating benign from malignant lesions. Current research has extensively examined PET/CT parameters such as Maximum Standardized Uptake Value (SUVmax) and the ratio of tumor SUVmax to liver SUVmax (T/L) in distinguishing benign from malignant adrenal masses [16]. However, a universally accepted standard has yet to be established, and false-positive and false-negative results remain a concern [11]. While histopathological examination remains the gold standard for accurate diagnosis of any mass, it can also lead to unnecessary anxiety, overtreatment, and potential complications such as hypertensive crises and tumor dissemination [11].

Radiomics can be defined as the objective and quantitative characterization of tumor phenotypes by converting medical images into a large set of quantitative image features using computational algorithms, thereby revealing tumor characteristics that are imperceptible to the naked eye. By applying statistical and/or machine learning methods for quantitative analysis, radiomics aims to enhance diagnostic accuracy and facilitate predictive modeling [17,18]. This approach holds significant potential for personalized cancer treatment [19,20,21]. Previous studies have demonstrated various applications of radiomics. Winkelmann MT et al. reported that Random Forest based on dual-energy CT achieved an AUC of 0.83 for distinguishing adrenal adenomas from metastases [22]. O’Shea et al. developed a single-phase, contrast-enhanced, CT-based nomogram incorporating age, prior malignancy history, and radiomic features, which showed excellent discriminatory performance (AUC = 0.904) in the validation cohort [23]. Stanzione et al. confirmed that MRI out-of-phase (OP) imaging radiomics can differentiate benign adrenal tumors from primary and metastatic malignancies [18]. However, to our knowledge, no previous studies have explored the application of radiomics features derived from [18F]Fluorodeoxyglucose (FDG) PET/CT in differentiating adrenal metastases for patients with extrarenal solid malignant tumors. Therefore, this study aims to explore the diagnostic efficacy of [18F]FDG PET/CT-based radiomics in identifying adrenal metastases and to compare it with that of conventional PET/CT parameters.

## 2. Materials and Methods

### 2.1. Patients

**Figure 1:**
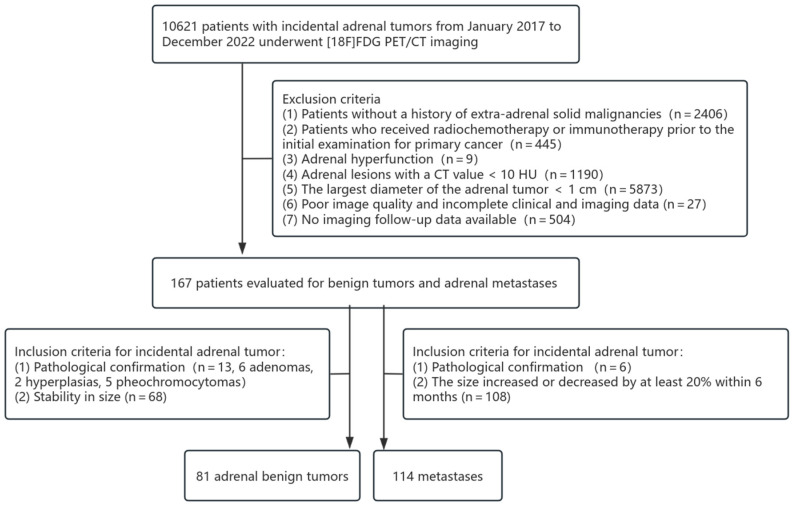
Patient selection flowchart for model development cohort.

**Figure 2:**
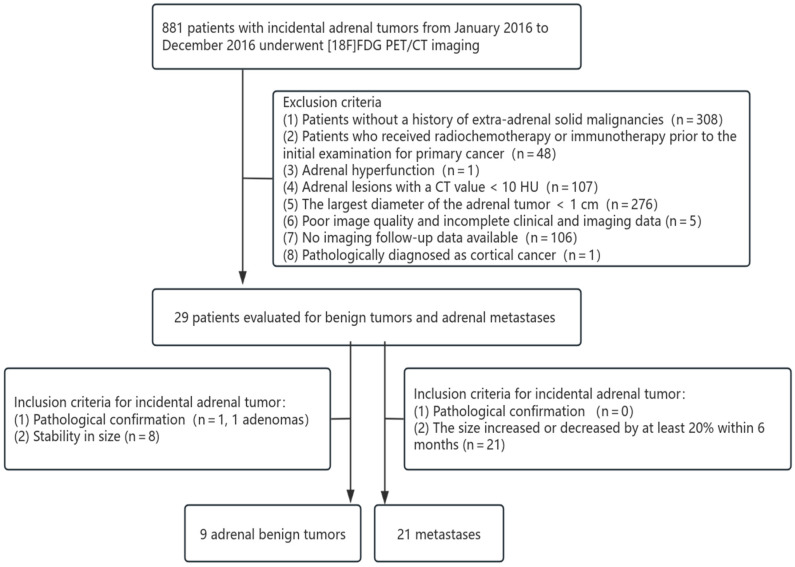
Patient selection flowchart for external validation cohort.

This study is a retrospective analysis. The data utilized, including medical records and imaging materials, have undergone complete de-identification to ensure that patient identities cannot be traced. Furthermore, this study involves only the analysis of existing data and does not entail any interventions or additional examinations on the subjects. In accordance with the relevant provisions of China’s “Ethical Review Measures for Biomedical Research Involving Human Beings”, this retrospective study conducted by our institution meets the criteria for ethical exemption and therefore does not require an Institutional Ethics Committee. Written informed consent was obtained from each patient to publish this paper. From January 2017 to December 2022, a total of 167 patients (74 in the benign tumor group and 93 in the metastases group) who underwent [18F]FDG PET/CT imaging at Peking University Cancer Hospital for the evaluation of extrarenal solid tumors were included in this study. Clinical and routine PET/CT parametric models, radiomics models, and nomograms were constructed based on this cohort. For external validation, an additional 30 patients meeting the same inclusion and exclusion criteria (10 in the primary tumor group and 20 in the metastatic tumor group) were recruited from the same center between January 2016 and December 2016. The inclusion criteria for benign tumors were (1) the largest diameter of the adrenal tumor > 1 cm; (2) confirmation by puncture pathology or surgical pathology; and (3) during cancer treatment, no significant change in the size of adrenal lesions over at least a 1-year interval [24]. The inclusion criteria for metastases group were (1) the largest diameter of the adrenal tumor > 1 cm; (2) confirmation by puncture pathology or surgical pathology; and (3) during cancer treatment, the volume of adrenal nodules in the same patient increased or decreased by at least 20% within 6 months [24]. Exclusion criteria included (1) patients without a history of extra-adrenal solid malignancies; (2) patients who received radiochemotherapy or immunotherapy prior to the initial examination for primary cancer; (3) adrenal hyperfunction; (4) adrenal lesions with a CT value < 10 HU; (5) poor image quality, making tumor boundaries difficult to identify; (6) incomplete clinical and imaging data. The exclusion criteria for the included cases are summarized in Figure 1 and Figure 2.

### 2.2. [18F]FDG PET/CT Image Acquisition and Reconstruction

[18F]FDG is synthesized from 18F produced by the HM-20 medical cyclotron (Sumitomo Corporation, Tokyo, Japan), with a final product radiochemical purity exceeding 95%. Patients were required to fast for 6–8 h prior to the procedure, and their fasting blood glucose levels were maintained below 11.1 mmol/L. Following intravenous administration of [18F]FDG at a dose of 3.7 MBq/kg, patients rested quietly for 60 min before drinking 800–1000 mL of water to distend the gastrointestinal tract in preparation for PET/CT examination. The scanning range extended from the base of the skull to the mid-thigh. Two PET/CT scanners, the uMI780 (United Imaging Healthcare, Shanghai, China) and Siemens Biograph (Siemens Healthineers, Erlangen, Germany), were utilized for image acquisition. Both scanners had passed the harmonization evaluations according to the EARL2 standard. For low-dose non-enhanced CT scans performed on both machines, identical parameters were employed: 120 kV tube voltage, automatic tube current, 3–5 mm slice thickness, 0.8 pitch, and a matrix of 512 × 512. PET images were acquired in 3D mode; the uMI780 collected data over 4–5 bed positions, while the Siemens Biograph used continuous bed motion mode at a speed of 1.3 mm/s with a matrix of 200 × 200. Whole-body PET and PET/CT fusion images were generated using low-dose unenhanced CT data for attenuation correction and iterative reconstruction. Finally, high-resolution chest CT images were obtained during breath-hold, covering the region from the skull base to the abdomen, with consistent acquisition parameters: 120 kV tube voltage, 250 mA tube current, 2 mm slice thickness, 1 mm spacing, and a matrix of 512 × 512.

### 2.3. Image Interpretation

PET/CT image registration and display were conducted on the United Imaging workstation (uWS-MI, uWS-CT, United Imaging Medical Technology, Shanghai, China) and the Siemens workstation (Syngo.via VB20, MM Oncology, Siemens Healthineers, Erlangen, Germany). Two nuclear medicine physicians with a minimum of three years of experience independently interpreted the images. They measured and recorded the SUVmax of the adrenal tumor and T/L, ensuring that they avoided blood vessels and metastases in PET images. In axial CT images, they also measured and recorded the maximum diameter (Dmax) based on the largest cross-sectional area, the average CT value from either the largest section or the most heterogeneous section of the adrenal gland, and the position (unilateral or bilateral) of AIs. Finally, the average of the two physicians’ measurements was calculated and reviewed by a senior nuclear medicine physician.

### 2.4. Construct Models of Clinical and Conventional PET/CT Parameters

Univariate logistic regression analysis was conducted to evaluate clinical information and standard PET/CT parameters, identifying potential factors that could determine the source. Multivariate logistic regression analysis was subsequently applied to screen for factors with *p* < 0.05 from the univariate logistic regression results. Factors with *p* < 0.05 in the multivariate logistic regression were identified as independent predictors for determining the source of AIs. Diagnostic models based on clinical and standard PET/CT parameters were then constructed using these independent predictors.

### 2.5. Three-Dimensional Segmentation

**Figure 3:**
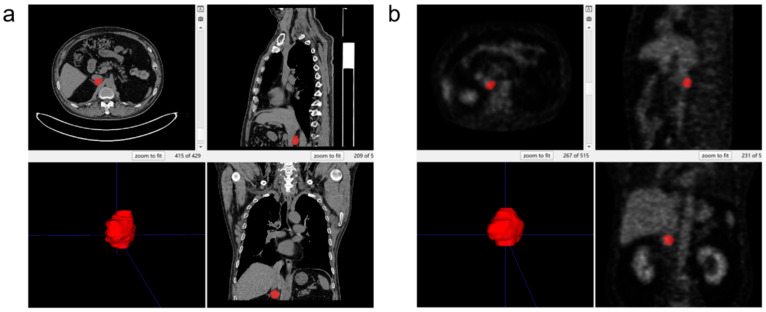
Region of interest diagram. (**a**) Lesion segmented on high-resolution CT; (**b**) lesion segmented on PET.

A nuclear medicine physician with three years of work experience imported PET images, whole-body low-dose CT images and high-resolution CT images into ITK-SNAP software (version 3.8.0; https://www.itksnap.org) respectively and manually delineated the region of interest (ROI) encompassing adrenal tumors on a layer-by-layer basis in the axial plane. In cases of disagreement between the initial physician and a senior reviewer, a third senior nuclear medicine physician provided assistance to reach a consensus. All segmented results, saved in NIfTI format, were prepared for subsequent image feature extraction and analysis. The segmented lesions are illustrated in Figure 3.

### 2.6. Feature Extraction and Screening

In the pre-processing stage, the ROI of CT and PET images was resampled to match the resolution of PET images and then standardized using z-score normalization, ensuring better alignment with PET images. The detailed preprocessing procedures are provided in the Supplementary Material. Radiomics features were extracted from PET and high-resolution CT images primarily using the Pyradiomics package in Python (version 3.7.11). A total of 200 quantitative features were extracted from each patient’s PET and CT images (100 from high-resolution CT and 100 from PET), covering six categories, as detailed in the Supplementary Material. Before feature selection, we implemented rigorous quality control, including inter-observer reproducibility assessment (ICC > 0.85 required for inclusion) and distribution normalization. Clinical data, routine parameters from high-resolution CT and PET images, and their radiomics features were included for feature selection via least absolute shrinkage and selection operator (LASSO) regression. We employed 100 iterations of 10-fold cross-validation to determine the optimal regularization parameter. By varying λ values, the mean square error (MSE) of the LASSO model was calculated, and the λ value corresponding to the smallest MSE was selected for final feature screening. Features with non-zero correlation coefficients were retained.

### 2.7. Constructed and Evaluated Models

Based on the selected features, ten machine learning methods were employed to construct models. First, AIs were randomly divided into a training test set and a validation set at a ratio of 7:3. To enhance model robustness, three rounds of five-fold cross-validation were performed on the training test set. The tuning was performed using a five-fold cross-validation repeated three times. We performed grid search within biologically plausible ranges—for instance, SVM’s C parameter was explored from 10⁻^3^ to 10^3^ in logarithmic steps. For computationally intensive parameters, Bayesian optimization was additionally applied to improve efficiency. The average accuracy of cross-validation for different models in the training test set and the accuracy, specificity, sensitivity, positive predictive value (PPV), and negative predictive value (NPV) in the validation set were calculated and compared to identify the optimal model. The area under the curves (AUC) of both the training test and validation sets were utilized to assess the model’s performance in differentiating between the benign tumor group and the metastases group. Figure S1 illustrates the workflow for constructing the ten radiomics models, while Figure S2 details the three rounds of five-fold cross-validation.

### 2.8. Draw and Evaluate Nomogram

The Rad_score was calculated as a linear combination of the radiomics features, weighted by their logistic regression coefficients. A nomogram was constructed by integrating the non-radiomics screened features with the Rad_score. The performance of the nomogram in distinguishing benign from metastatic AIs was evaluated using calibration curves derived from both the training test and validation sets. To assess the clinical utility of the nomogram, decision curve analysis (DCA) was performed to calculate the net benefit within the threshold probability range and to determine whether the model could provide clinical benefits to patients.

### 2.9. External Validation Protocol

All external validation cases underwent identical preprocessing and quality control procedures as the training set. Subsequently, the preprocessed data were separately input into the pre-trained radiomics model and clinical nomogram for parallel prediction. During the prediction process, both models were run in their original developed states without any modifications. The radiomics model automatically calculated positive probabilities and output results through Python scripts, while the nomogram allowed physicians to interactively input clinical parameters and visualize risk probabilities. To eliminate evaluation bias, all prediction results were recorded by independent researchers under blinded conditions and compared against the gold standard for analysis.

## 3. Results

A total of 167 patients were enrolled in this study. The benign tumor group comprised 43 males (58.1%) and 31 females (41.9%), with ages ranging from 40 to 81 years (mean ± SD: 63.20 ± 7.94). Of these, sixty-seven patients (90.5%) had unilateral Ais, and seven patients (9.5%) had bilateral AIs. The group included 44 lung cancer patients; the remaining non-lung cancer patients consisted of 11 cases of esophageal cancer, five cases of breast cancer, four cases each of gastric and colorectal cancer, two cases of liver cancer, and one case each of pancreatic, kidney, ovarian, and cervical cancer. The metastases group included 67 males (72.0%) and 26 females (28.0%), with ages ranging from 22 to 82 years (mean ± SD: 60.55 ± 10.10). Of these, 72 patients (77.4%) had unilateral AIs, and 21 patients (22.6%) had bilateral AIs. The group included 77 lung cancer patients; the remaining non-lung cancer patients consisted of four cases of gastric cancer, three cases each of esophageal and colorectal cancer, and one case each of gastrointestinal stromal tumor, gastrointestinal endocrine tumor, bile duct cancer, ampulla cancer, kidney cancer, and breast cancer.

In the benign tumor group, there were 81 adrenal lesions, of which 13 were confirmed pathologically (six adenomas, two hyperplasias, and five pheochromocytomas). In the metastases group, there were one hundred and fourteen adrenal lesions, with six confirmed pathologically and the remainder confirmed by imaging follow-up. The basic characteristics of the patients are summarized in Table 1.

There were statistically significant differences between the two groups in gender (*p* = 0.043), the site of adrenal incidentalomas (AIs) (*p* = 0.036), and whether the primary lesion was lung cancer (*p* = 0.001). Additionally, significant differences were observed in Dmax, CT value, SUVmax, and T/L ratio of adrenal tumors between the two groups (all *p* < 0.001). However, there was no statistically significant difference in age (*p* = 0.066).

Lung cancer, Dmax, bilateral AIs, CT value, SUVmax, and T/L ratio were included in univariate and multivariate logistic regression analyses. The results indicated that lung cancer, Dmax, CT value, and T/L ratio were independent risk factors for differentiating benign from metastatic adrenal incidentalomas (all *p* < 0.05) (Table 2). Based on these four independent risk factors, eight diagnostic models were constructed (Table 3). Among these models, both the T/L ratio diagnostic model (AUC: 0.920) and the model combining clinical data with PET/CT (AUC: 0.919) demonstrated higher efficacy. Specifically, sensitivity, specificity, and accuracy of the T/L diagnostic model were 0.925, 0.824, and 0.844, respectively, while those of the combined clinical and PET/CT diagnostic model were 0.849, 0.892, and 0.844, respectively.

A total of 105 CT and 105 PET imaging features were extracted in this study. These features include 18 first-order statistical features, 14 shape features, 22 Gray Level co-occurrence Matrix (GLCM) features, 14 Gray Level Dependence Matrix (GLDM) features, 16 Gray Level Size Zone Matrix (GLSZM) features, five Neighboring gray tone difference matrix (NGTDM) features, and 16 Gray-Level Run-Length Matrix (GLRLM) features. Lasso–Cox regression with the optimal penalty parameter λ was subsequently employed to further screen clinical, imaging, and radiomics features. The optimal penalty parameter λ and the corresponding characteristic heatmap are presented in Figures S3 and S4. Ultimately, four clinical and imaging features and eight radiomics features were selected for model construction. The performance of the ten radiomics models is summarized in Table 4.

**Figure 4:**
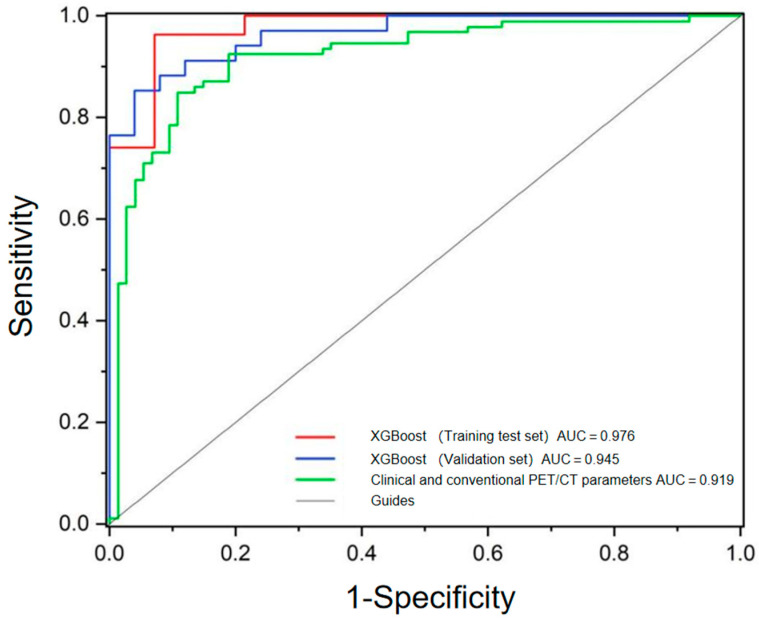
Comparative ROC analysis of radiomics and conventional PET/CT models.

The results demonstrated that XGBoost had the highest overall accuracy in both the training (0.883) and validation sets (0.932), along with excellent sensitivity (0.930), specificity (0.960), PPV (0.970), and NPV (0.890). LightGBM and AdaBoost also exhibited strong performance, achieving validation accuracies of 0.915, with LightGBM showing particularly high specificity (0.960) and PPV (0.970). In contrast, Decision Tree and KNN models underperformed, with validation accuracies of only 0.797 and 0.831, respectively. Notably, GaussianNB achieved the highest specificity (0.960) and PPV (0.970), albeit with moderate sensitivity (0.850), suggesting its potential utility in scenarios where minimizing false positives is critical. Conversely, GDBT and LR exhibited high sensitivity (0.940) but relatively lower specificity, making them suitable for applications prioritizing the detection of true positives. These findings highlight XGBoost as the most robust and generalizable model, with AUC values of 0.976 and 0.945 for the training test set and validation set, respectively. Moreover, it demonstrated superior performance compared to the model constructed by clinical and conventional PET/CT parameters. The ROCs of these two models are shown in Figure 4. However, the Delong test revealed no statistically significant differences among the three AUCs (all *p* > 0.05).

The Rad_score quantifies tumor characteristics using radiomics features. The equations are now written as

$$\begin{array}{c} \mathrm{Rad}\_\mathrm{score}=\sum_{i=1}^{n}\omega_{i}\cdot f_{i}+b \end{array}$$

In the formula, $\omega_{i}$ is the regression coefficient of the *i*-th radiomics feature, $f_{i}$ is the standardized value of the *i*-th radiomics feature, and b is the intercept term to adjust the baseline score.

Specifically, in this study,

Rad_score = −0.34 × PET_original_glcm_InverseVariance

−0.32 × original_shape_Flatness

−0.21 × PET_original_glszm_GrayLevelNonUniformityNormalized

−0.18 × PET_original_ngtdm_Coarseness

−0.22 × original_glcm_InverseVariance

+0.30 × original_glcm_JointEnergy

+0.28 × PET_original_glrlm_RunEntropy

−0.25 × PET_original_shape_Elongation + 0.41

Similarly, the Nomo_score integrates clinical parameters, conventional PET/CT metrics, and Rad_score for comprehensive prediction. The equations are now written as

Nomo_score = 0.50 × primary_cancer + 0.12 × tumor_SUVmax + 0.09 × CT_value − 0.04 × age + 0.82 × Rad_score − 1.25

**Figure 5:**
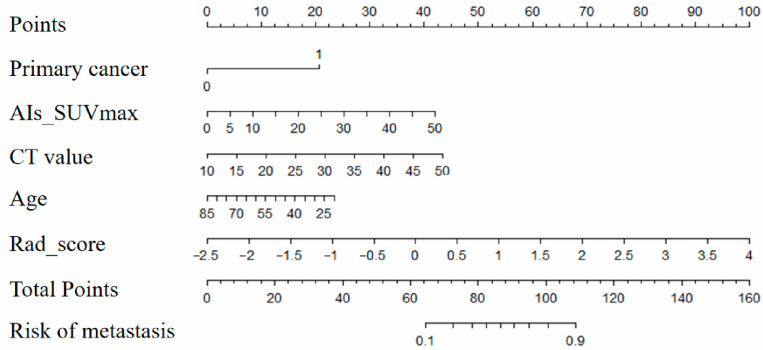
Nomogram. AIs, adrenal incidentalomas; SUVmax, Maximum Standardized Uptake Value.

Figure 5 illustrates the nomogram. The calibration curve demonstrates good agreement between the nomogram-predicted values and the observed true values. (Figure S5). The C-index values for the training set and validation set were 0.938 and 0.906, respectively. As shown in Figure S6, the nomogram provided a higher net benefit across the whole range of threshold probabilities (0% to 100%) compared with the treat-all and treat-none strategies. In the validation cohort, for threshold probabilities between 40% and 80%, the nomogram consistently achieved positive net benefit values exceeding 0.3, indicating improved clinical decision-making compared to either indiscriminate treatment or omission. These results support the practical utility of the nomogram in identifying patients with adrenal metastases who may benefit from further intervention or monitoring, thus aiding in risk stratification and treatment planning.

The clinical and imaging characteristics of the cases in the external validation group are summarized in Table 5. In the benign tumor group, there were seven patients with lung cancer, one patient with esophageal carcinoma, and one patient with liposarcoma. All AIs were unilateral, and pathology confirmed one case of adenoma. In the metastases group, there were fifteen patients with lung cancer, one patient with esophageal cancer, one patient with kidney cancer, one patient with breast cancer, and two patients with colorectal cancer. One patient had bilateral AIs, and all 21 AI lesions were confirmed by imaging follow-up.

**Figure 6:**
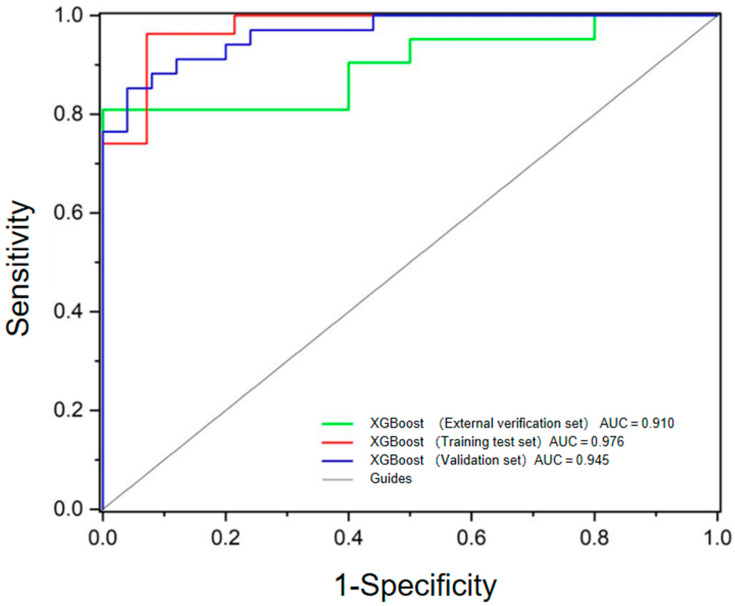
ROC analysis of XGBoost model for the training test set, validation set, and external validation set.

The XGBoost model and the nomogram were externally validated on 30 lesions, with prediction results shown in Table S1. The models were consistent in 25 (25/30) lesions. Among these, one lesion (1/25) was diagnosed as false negative by both the XGBoost model and the nomogram, while the remaining predictions were consistent with true values. In the XGBoost model, three lesions were diagnosed as false negatives, resulting in an accuracy, sensitivity, specificity, PPV, and NPV of 0.900, 0.860, 1.000, 1.000, and 0.750, respectively. For the nomogram, three lesions were diagnosed as false positives and one as a false negative, leading to an accuracy, sensitivity, specificity, PPV, and NPV of 0.870, 0.952, 0.667, 0.870, and 0.857, respectively. Figure 6 presents the ROC curves for the XGBoost model across the training test set, validation set, and external validation set, with an AUC of 0.910 in the external validation set.

**Figure 7:**
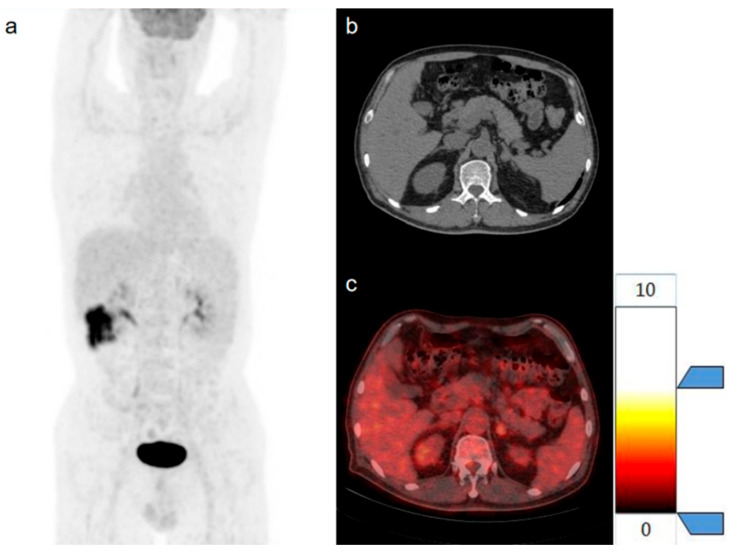
Patient No. 6 (**a**) The maximum density projection (MIP), (**b**) the high-resolution CT, and (**c**) the PET/CT.

**Figure 8:**
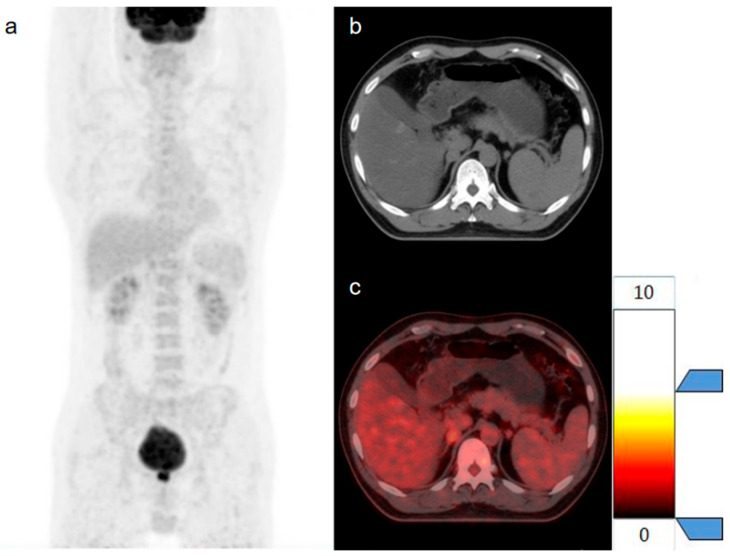
Patient No. 15 (**a**) The maximum density projection (MIP), (**b**) the high-resolution CT, and (**c**) the PET/CT.

Patient No. 6 (Figure 7) was a 61-year-old male with colon cancer, and Patient No. 15 (Figure 8) was a 45-year-old male with rectal cancer. AIs were incidentally detected during [18F]FDG PET/CT examinations in both patients, with no other abnormal findings within the scan range except for the AIs. In Patient No. 6, a soft tissue density mass measuring 1.7 cm in maximum diameter was observed in the left adrenal gland, with a CT value of 34 HU, SUVmax of 3.0, and a T/L ratio of 1.25. Pathological biopsy confirmed this lesion as an adenoma, and the final staging was T3N0M0. In Patient No. 15, a soft tissue density mass measuring 1.9 cm in maximum diameter was observed in the right adrenal gland, with a CT value of 40 HU, SUVmax of 3.9, and T/L ratio of 1.15. Follow-up imaging confirmed this lesion as a metastatic tumor, and the final staging was T2N0M1. Based solely on clinical information and conventional PET/CT parameters, it is challenging to differentiate between benign and metastatic AIs. However, the predictions of the radiomics model were consistent with the actual diagnoses, thereby facilitating accurate staging for these patients.

## 4. Discussion

To date, no studies have been reported domestically or internationally on PET/CT-based radiomics specifically aimed at differentiating adrenal metastases in patients with extrarenal malignant solid tumors. In our study, lung cancer (primary malignant tumor), and CT value, maximum diameter, and T/L were found to be independent risk factors for AIs, consistent with previous reports [25,26,27,28]. Among patients with adrenal metastatic tumors, up to 39–47% of primary malignancies are lung cancers [29,30]. In this study, lung cancer accounted for 82.8% of primary cancers in adrenal metastatic tumor patients, significantly higher than previously reported rates. This discrepancy may be attributed to the large number of lung cancer patients in our hospital and potential selection bias. It was reported that the sensitivity and specificity for detecting adrenal metastases when the maximum adrenal diameter was >2.2 cm and CT value was >20 HU were 73.1%, 78.5% and 95%, 83.3%, respectively [28], which were lower than those observed in this study. This discrepancy may be attributed to the focus on adrenal high-density nodules (CT value > 10 HU) in our study. Furthermore, recent studies have indicated a relatively high false positive and false negative rate in CT diagnosis of adrenal metastases [31]. Although statistically significant differences were found in AIs’ SUVmax and T/L between the two groups, only T/L emerged as an independent factor. Our study demonstrated that T/L exhibited a stronger correlation with adrenal metastasis. Furthermore, among the four independent risk factors, T/L achieved the highest diagnostic efficacy, with an AUC of 0.920 (95% CI: 0.878, 0.963), yielding a sensitivity of 0.925 and specificity of 0.824, although the accuracy (0.844) was slightly lower than previously reported [32]. The optimal T/L cutoff value identified in this study was 1.4. Furthermore, consistent with previous findings, although men are more frequently diagnosed with metastatic adrenal tumors compared to patients with benign adrenal tumors [33], metastasis did not emerge as an independent risk factor. This may be attributed to the higher incidence of lung cancer in men [34], leading to a skewed gender ratio between the two groups.

In the section on building the radiomics model and nomogram, we ultimately selected four clinical and imaging features and eight radiomic features for inclusion. These features included primary tumor, age, CT value and SUVmax of AIs, and radiomic features such as original_shape_Flatness, original_glcm_InverseVariance, and original_glcm_JointEnergy extracted from high-resolution CT, as well as original_shape_Elongation, original_glszm_GrayLevelNonUniformityNormalized, original_ngtdm_Coarseness, original_glcm_InverseVariance, and original_glrlm_RunEntropy extracted from PET. Compared with other models, XGBoost demonstrated the highest diagnostic efficiency. This is attributed to the ability of radiomic features to deeply extract hidden information within lesions, thereby reflecting various aspects of tumor heterogeneity. The study by Caruso et al. [35] also confirmed that malignant tumors exhibit higher heterogeneity. Additionally, shape features describing the smoothness and elongation of the ROI may be associated with tumor aggressiveness, although their acquisition can be subjectively influenced by the physician who delineates the ROI. Studies have shown that combining the constructed PET/CT radiomics model with manual diagnosis can significantly reduce the false positive rate and improve diagnostic accuracy compared to manual diagnosis alone [36]. Nomograms can transform graph features into statistical prediction models, estimating individualized risks based on each feature, which can help identify and stratify patients for participation in clinical trials [37]. The machine learning classifiers were trained exclusively on radiomics features to optimize predictive accuracy and explore the comparative performance of various algorithms in identifying adrenal metastases. These models operate in high-dimensional feature space and are useful for automated analysis pipelines or AI-assisted diagnostic systems. In contrast, the nomogram was developed by integrating the ML-derived Rad_score with selected clinical and conventional imaging features. This composite model was visualized in a nomogram format to enhance clinical interpretability. The nomogram offers a user-friendly tool for individualized risk assessment in clinical settings, where decision support tools need to be both accessible and understandable to physicians. Therefore, the nomogram complements the ML classifiers by providing a practical bridge between advanced computational models and real-world clinical decision-making. It is a simple, easy-to-understand, and user-friendly clinical decision-making tool [38], widely applied in personalized prognosis assessment for cancer patients, including lung cancer [39,40], breast cancer [41], pancreatic cancer [42], colon cancer [43,44], and prostate cancer [45]. In our study, the decision curve analysis confirmed the clinical utility of the nomogram. In external validation, XGBoost achieved high diagnostic efficiency, with an AUC value of 0.910 and specificity and PPV of 100%. However, while the sensitivity and NPV of the nomogram were higher than those of XGBoost, its accuracy, specificity, and PPV were less satisfactory. Notably, among the thirty adrenal tumors, twenty-five cases showed agreement between XGBoost and nomogram predictions, with one false-negative case. Therefore, when both models agreed, the accuracy rate was 96%. When XGBoost predicted negative but the nomogram predicted positive, the positive result should be preferred. Conversely, if the predictions were reversed, the negative result should be preferred.

However, certain limitations should be acknowledged. Firstly, this is a single-center, small-sample retrospective study, which may introduce selection bias and limit the generalizability of the model due to the constrained dataset. Secondly, not all adrenal lesions were histologically confirmed because of the potential risks and contraindications associated with biopsy or surgery, leading to a relatively low proportion of pathologically confirmed cases, which reflects and is also in line with the current clinical reality. Thirdly, 3D manual ROI segmentation is time-consuming, complex, and susceptible to inter-observer variability. In this study, three nuclear medicine physicians reviewed the ROIs, which mitigated individual differences to some extent, but future research should focus on developing automatic segmentation methods with high reliability and reproducibility. Fourthly, our study exclusively utilized three-dimensional regions of interest (3D VOI). While previous studies have reported that 3D VOI better reflects tumor heterogeneity compared to two-dimensional regions of interest (2D ROI) [46], 2D ROI offers operational simplicity, potentially making it more feasible for routine clinical use and easier to promote. Lastly, external validation was conducted using patients from our center at different time periods, without including patients from other centers. Future studies should aim to conduct multi-center trials to build larger, more diverse datasets for evaluating the proposed model. Prospective studies are also necessary to verify the robustness and effectiveness of our model.

## 5. Conclusions

Adrenal incidentalomas are frequently detected in cancer patients, and accurate differentiation between benign and malignant lesions is crucial for clinical staging and treatment planning. Radiomics has emerged as a promising approach in this field. Our PET/CT-based radiomics model demonstrated excellent diagnostic performance, achieving AUC values of 0.945 in internal validation and 0.910 in external validation for distinguishing benign non-lipomatous adrenal tumors from metastatic lesions. The developed nomogram effectively integrates radiomic features with clinical parameters into a visual scoring system, providing clinically interpretable risk stratification while maintaining diagnostic accuracy (external validation accuracy: 0.893). Further multicenter validation with larger cohorts is warranted to confirm its clinical utility.

## Figures and Tables

**Table 1:** Clinical and radiological characteristics of the model development cohort.

Parameters	Benign Tumor Group	Metastases Group	*p*
Number of patients	74	93	
Number of tumors	81	114	
Age	63.20 ± 7.94	60.55 ± 10.10	0.066
Gender			0.043 *
Female	31 (41.9%)	26 (28.0%)	
Male	43 (58.1%)	67 (72.0%)	
Maximum diameter (cm)	1.90 ± 0.58	2.55 ± 1.28	<0.001 *
Location			0.036 *
Unilateral	67 (90.5%)	72 (77.4%)	
Bilateral	7 (9.5%)	21 (22.6%)	
CT value(HU)	23.36 ± 8.73	32.36 ± 6.16	<0.001 *
SUVmax	2.8 (2.00, 3.60)	7.5 (5.40, 10.93)	<0.001 *
T/L	0.91 (0.67, 1.2)	2.83 (1.84, 4.02)	<0.001 *
Primary tumor			0.001 *
Lung cancer	44, 59.5%	77, 82.8%	
Non-lung cancer	30, 40.5%	16, 17.2%	

SUVmax, Maximum Standardized Uptake Value; T/L, tumor SUVmax/liver SUVmax; HU, Hounsfield units; * means *p* < 0.05.

**Table 2:** Univariate and multivariate logistic regression analysis results.

Parameters	Univariate Analysis		Multivariate Analysis	
	OR (95%CI)	*p*	OR (95%CI)	*p*
Lung cancer	3.281 (1.612, 6.680)	0.001	6.429 (2.190, 18.873)	0.001 *
Male	1.858 (0.973, 3.547)	0.061		
Dmax (cm)	2.249 (1.463, 3.457)	<0.001	2.099 (1.064, 4.142)	0.033 *
Bilateral	2.622 (1.043, 5.596)	0.041	3.432 (0.782, 15.068)	0.102
CT value (HU)	1.155 (1.101, 1.213)	<0.001	1.138 (1.069, 1.212)	<0.001 *
SUVmax	1.608 (1.366, 1.894)	<0.001	0.694 (0.401, 1.204)	0.194
T/L	3.876 (2.443, 6.148)	<0.001	5.402 (1.050, 27.802)	0.044 *

In univariate analysis, parameters with OR > 1 and *p* < 0.05 were screened for multivariate analysis. In multivariate analysis, parameters with OR > 1 and *p* < 0.05 were used as independent risk factors to construct the diagnostic models. Dmax, maximum diameter; T/L, tumor SUVmax/liver SUVmax; HU, Hounsfield units; * means *p* < 0.05.

**Table 3:** Predictive performance of independent risk factor-based models.

Models	AUC (95%CI)	Sensitivity	Specificity	Accuracy
Primary tumor	0.617 (0.530, 0.704)	0.828	0.405	0.641
Dmax	0.664 (0.582, 0.745)	0.742	0.514	0.623
CT value	0.783 (0.709, 0.856)	0.860	0.649	0.766
T/L	0.920 (0.878, 0.963)	0.925	0.824	0.844
CT value + Dmax	0.835 (0.774, 0.896)	0.742	0.797	0.749
CT value + Dmax + T/L	0.914 (0.867, 0.960)	0.892	0.824	0.844
Primary tumor + CTvalue + Dmax	0.870 (0.816, 0.926)	0.849	0.811	0.814
Primary tumor + CT value + Dmax + T/L	0.919 (0.874, 0.963)	0.849	0.892	0.844

Dmax, maximum diameter; T/L, tumor SUVmax/liver SUVmax.

**Table 4:** Performance of different radiomics models.

Model	Training Test Set	Validation Set				
	Accuracy	Accuracy	Sensitivity	Specificity	PPV	NPV
RF	0.811	0.864	0.900	0.830	0.840	0.890
AdaBoost	0.824	0.915	0.910	0.920	0.940	0.880
KNN	0.856	0.831	0.790	0.880	0.900	0.760
GaussianNB	0.816	0.898	0.850	0.960	0.970	0.830
Decision Tree	0.787	0.797	0.820	0.760	0.820	0.760
GDBT	0.855	0.898	0.940	0.840	0.890	0.910
LightGBM	0.878	0.915	0.880	0.960	0.970	0.860
XGBoost	0.883	0.932	0.930	0.960	0.970	0.890
LR	0.826	0.898	0.940	0.840	0.890	0.910
SVM	0.873	0.864	0.880	0.840	0.880	0.840

RF, Random Forest; AdaBoost, Adaptive Boosting; KNN, K-nearest Neighbor; GaussianNB, Gaussian Naive Bayes; GDBT, Gradient Boosting Decision Tree; LightGBM, Light Gradient Boosting Machine; XGBoost, eXtreme Gradient Boosting; LR, logistic regression; SVM, Support Vector Machine; PPV, positive predictive value; NPV, negative predictive value.

**Table 5:** Clinical and radiological characteristics of the external validation cohort.

Parameters.	Benign Tumors Group	Metastases Group
Number of patients	9	20
Age	63.89 ± 10.62	56.48 ± 11.30
Gender		
Male	7 (77.8%)	17 (85.0%)
Female	2 (22.2%)	3 (15.0%)
Location		
Unilateral	9	19
Bilateral	0	1
Dmax(cm)	1.83 ± 0.95	2.33 ± 1.05
CT(HU)	27.00 ± 6.61	29.76 ± 5.33
SUVmax	3.80 (2.85, 5.20)	7.30 (6.05, 9.70)
T/L	1.35 (0.92, 1.85)	2.30 (1.70, 3.47)
Primary tumor		
Lung cancer	7, 77.8%	15, 75.0%
Non-lung cancer	2, 22.2%	5, 25.0%

Dmax, maximum diameter; SUVmax, Maximum Standardized Uptake Value; T/L, tumor SUVmax/liver SUVmax; HU, Hounsfield units.

## Data Availability

The original contributions presented in this study are included in the article/Supplementary Material. Further inquiries can be directed to the corresponding author.

## Supplementary Materials

The following supporting information can be downloaded at https://www.mdpi.com/article/10.3390/diagnostics15111356/s1; Figure S1. Flowchart for constructing radiomics models; Figure S2. Flowchart of three five-fold cross-validation processes; Figure S3. The optimal penalty parameter λ; Figure S4. Characteristic heatmap; Figure S5. Nomogram calibration curves; Figure S6. Decision curve analysis of nomogram; Table S1. Predictive performance of the XGBoost model and nomogram in external validation.

## Abbreviations

**AIs**	**a**drenal incidentalomas
**[18F]FDG**	**[**18F]Fludeoxyglucose
**PET/CT**	**p**ositron emission tomography combined with computed tomography
**SUVmax**	**M**aximum Standardized Uptake Value
**T/L**	**t**umor SUVmax/liver SUVmax
**H** **U**	**H**ounsfield units
**Dmax**	**m**aximum diameter
**ROI**	**r**egion of interest
**LASSO**	**t**he least Absolute shrinkage and selection operator
**MSE**	**m**ean square error
**ROC**	**R**eceiver Operating Characteristic Curve
**AUC**	**a**rea under the curve
**DCA**	**d**ecision curve analysis
**APW**	**A**bsolute Percentage Washout
**RPW**	**R**elative Percentage Washout
**RF**	**R**andom Forest
**AdaBoost**	**A**daptive Boosting
**KNN**	**K**-nearest Neighbor
**GaussianNB**	**G**aussian Naive Bayes;
**GDBT**	**G**radient Boosting Decision Tree
**LightGBM**	**L**ight Gradient Boosting Machine
**XGBoost**	**e**Xtreme Gradient Boosting
**LR**	**l**ogistic regression
**SVM**	**S**upport Vector Machine
**PPV**	**p**ositive predictive value
**NPV**	**n**egative predictive value
**GLCM**	**G**ray Level co-occurrence Matrix
**GLDM**	**G**ray Level Dependence Matrix
**GLSZM**	**G**ray Level Size Zone Matrix
**NGTDM**	**N**eighboring gray tone difference matrix
**GLRLM**	**G**ray-Level Run-Length Matrix
**MIP**	**m**aximum Density projection
