# Supplementary material for: The Value of PET/CT-Based Radiomics in Predicting Adrenal Metastases in Patients with Cancer
Source: Diagnostics (Basel). 2025 May 28;15(11):1356. doi: 10.3390/diagnostics15111356 (PMC12154074; doi:10.3390/diagnostics15111356)
Supplement: Supplementary file 1 [file diagnostics-15-01356-s001.zip › diagnostics-3540429-supplementary.pdf]

The detailed preprocessing procedures:

To ensure data consistency and standardized model input, all imaging data underwent a uniform preprocessing pipeline. To achieve spatial alignment across multimodal inputs, all CT and PET images were resampled accordingly.

In the dataset from Peking University Cancer Hospital, high-resolution CT images had a voxel spacing of [1, 0.79, 0.79] mm and an in-plane matrix size of 512 × 512. The low-dose CT images used for attenuation correction had a voxel spacing of [3, 1.17, 1.17] mm and the same matrix size of 512 × 512. PET images had a voxel spacing of [4, 4, 4] mm and a matrix size of 144 × 144.

All PET images were resampled using linear interpolation to match the voxel spacing of the corresponding low-dose CT images.

The details of 200 features:

All radiomics features were extracted using PyRadiomics from segmented CT and PET volumes of interest. The features were categorized as follows:

- CT Radiomics Features (n=100 total)
  - First-order statistics (n=18): Quantifying intensity distributions (e.g., energy, entropy, kurtosis).
  - Shape-based (n=14): 3D tumor morphology (e.g., sphericity, surface area, volume).
  - Texture features (n=68):
    - Gray-level co-occurrence matrix (GLCM, n=22): Spatial relationships of voxel intensities.
    - Gray-level run-length matrix (GLRLM, n=16): Quantifying pixel runs.
    - Gray-level size zone matrix (GLSZM, n=16): Homogeneous zone sizes.
    - Gray-level dependence matrix (GLDM, n=14): Intensity dependencies.
- PET Radiomics Features (n=100 total)
  - First-order statistics (n=18): Quantifying intensity distributions (e.g., energy, entropy, kurtosis).
  - Shape-based (n=14): 3D tumor morphology (e.g., sphericity, surface area, volume).

- Texture features (n=68):
  - Gray-level co-occurrence matrix (GLCM, n=22): Spatial relationships of voxel intensities.
  - Gray-level run-length matrix (GLRLM, n=16): Quantifying pixel runs.
  - Gray-level size zone matrix (GLSZM, n=16): Homogeneous zone sizes.
  - Gray-level dependence matrix (GLDM, n=14): Intensity dependencies.

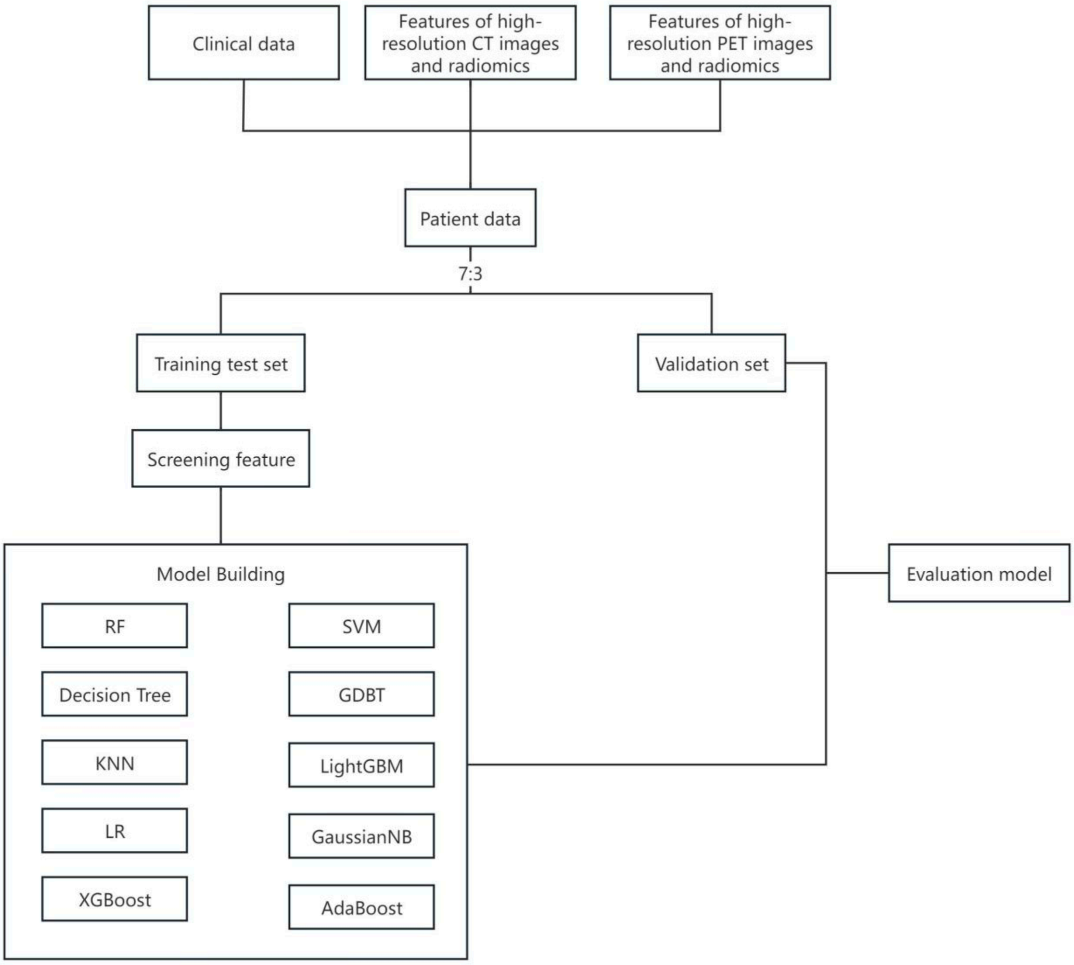

Figure S1. Flowchart for constructing radiomics models. RF, Random Forest; AdaBoost, Adaptive Boosting; KNN, K-nearest Neighbor; GaussianNB, Gaussian Naive Bayes; GDBT, Gradient Boosting Decision Tree; LightGBM, Light Gradient Boosting Machine; XGBoost, eXtreme Gradient Boosting; LR, Logistic Regression; SVM, Support Vctor Machine.

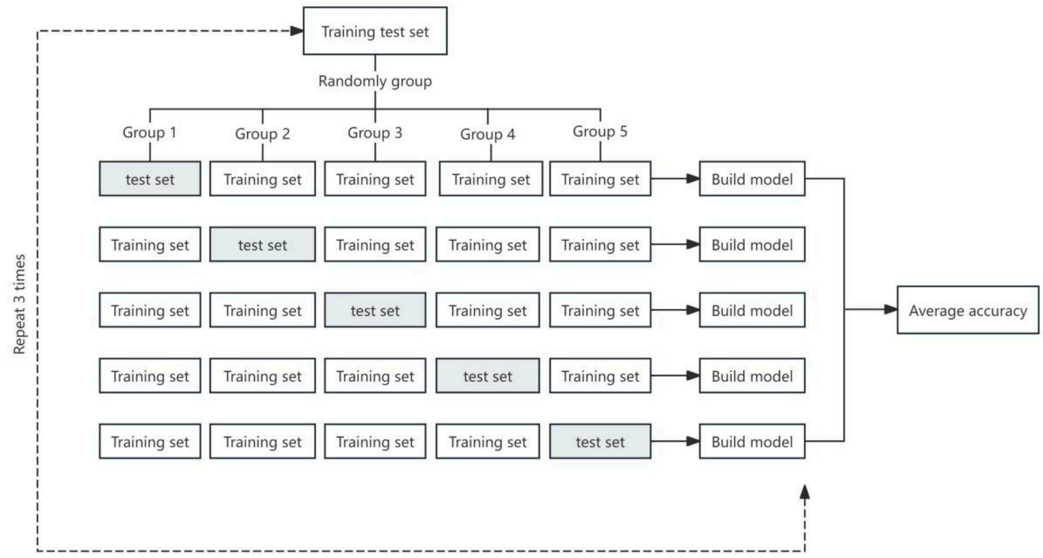

Figure S2. Flowchart of three five-fold cross-validation processes.

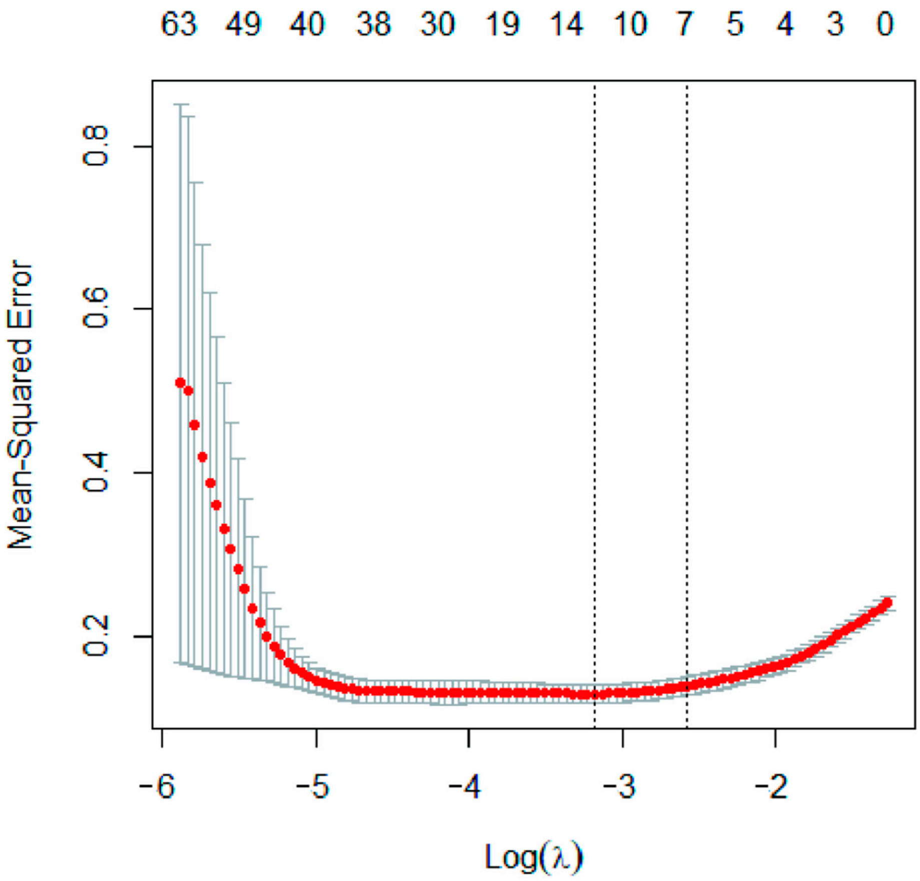

Figure S3. The optimal penalty parameter λ.

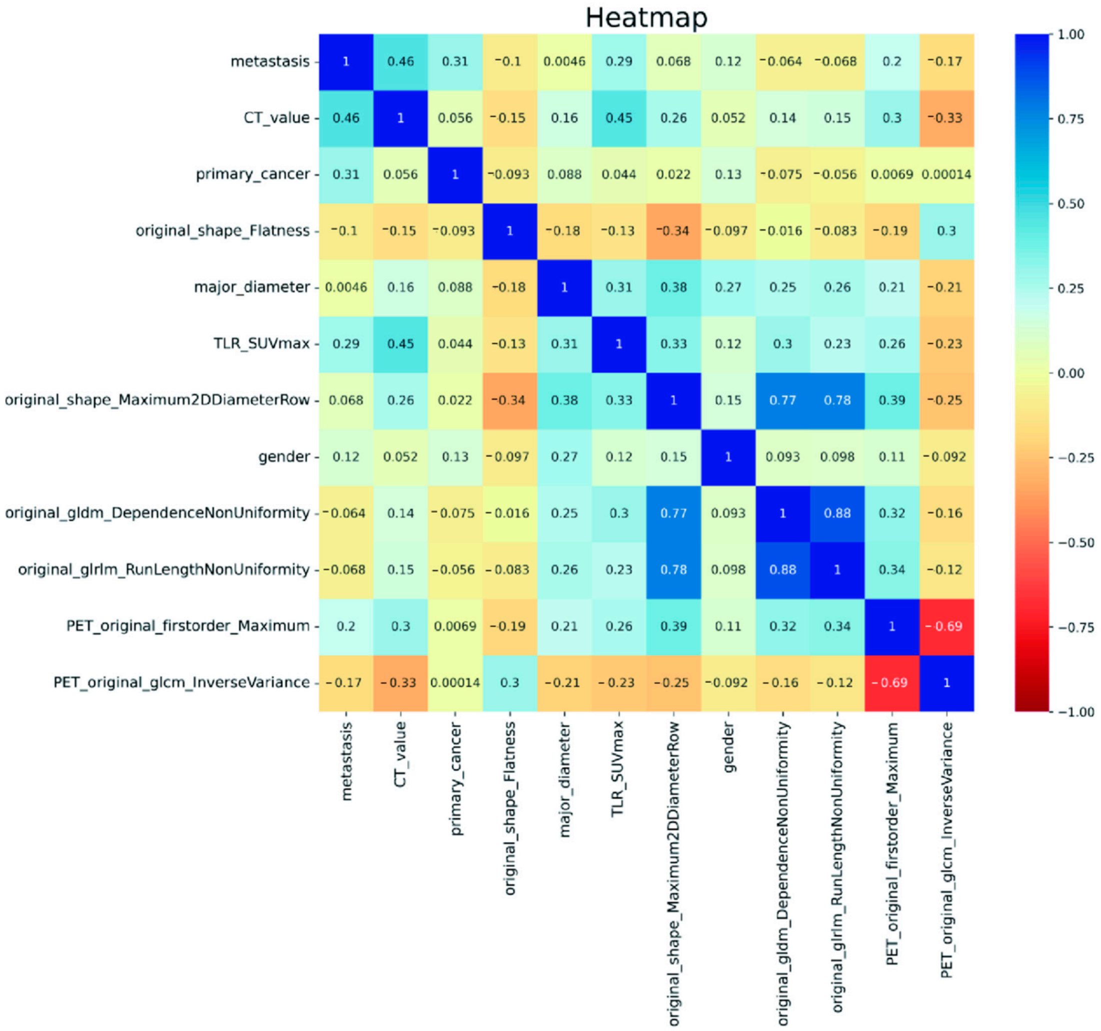

Figure S4. Characteristic heatmap

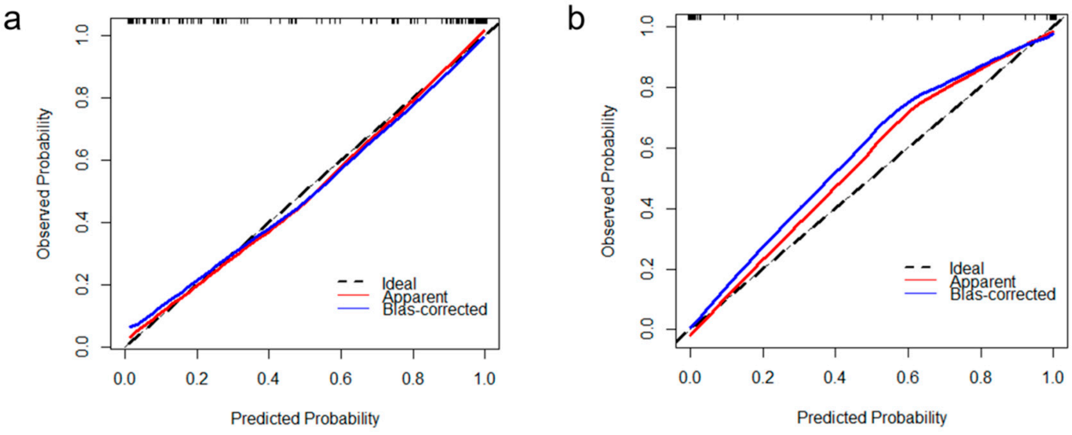

Figure S5. Nomogram calibration curves. a: The radiomics nomogram calibration curves in training test set；b: The radiomics nomogram calibration curves in validation set.

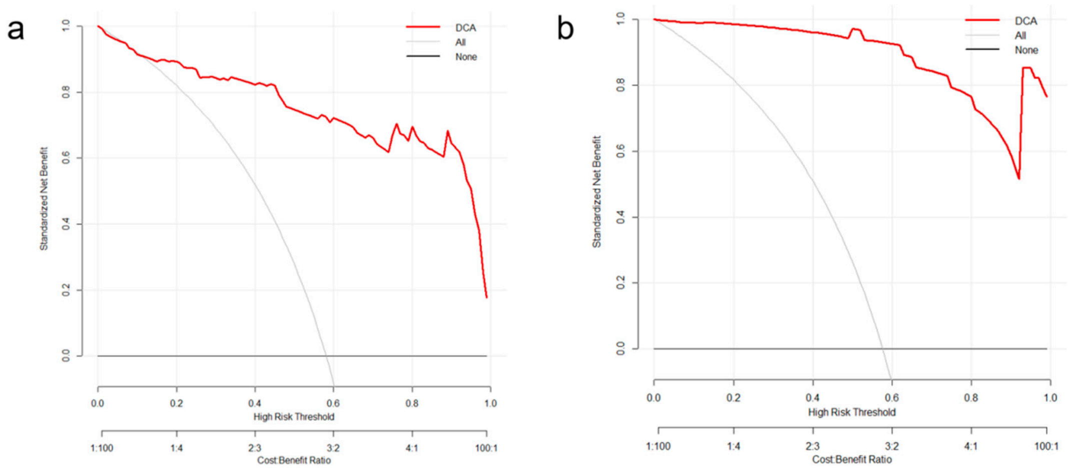

Figure S6. Decision curve analysis of nomogram a: Decision curve analysis of nomogram in training test set；b: Decision curve analysis of nomogram in validation set.

Table S1. Predictive performance of the XGBoost model and nomogram in external validation

Patient	Benign/ Metastasis	XGBoost	Nomogram
1	0	0.3762	0.9*
2	0	0.004512	0.3
3	0	0.2679	0.8*
4	0	0.003127	0.4
5	0	0.00856	0.3
6	0	0.06478	0.3
7	0	0.075998	0.6*
8	0	0.003828	0.3
9	0	0.01347	0.3
10	1	0.9916	0.6
11	1	0.9817	0.9
12	1	0.9936	0.9
13	1	0.9964	0.9
14	1	0.9989	0.9
15	1	0.8964	0.8
16	1	0.9861	0.9
17	1	0.9981	0.9
18	1	0.993	0.9
19	1	0.9856	0.9
20	1	0.9722	0.9
21	1	0.9928	0.9
22	1	0.9834	0.9
23L	1	0.9727	0.8
23R	1	0.8781	0.7
24	1	0.01582*	0.2*
25	1	0.0337*	0.8
26	1	0.9993	0.9
27	1	0.999	0.9
28	1	0.9027	0.9
29	1	0.001611*	0.8

0 and 1 indicate benign and metastatic tumors, respectively. 23L and 23R denote the left and right adrenal incidentalomas of Patient 23, respectively. An asterisk (*) indicates that the value does not match the ground truth.
